# Ultra-processed foods in a rural Ecuadorian community: associations with child anthropometry and bone maturation

**DOI:** 10.1017/S0007114523000624

**Published:** 2023-11-14

**Authors:** Emmanuel A. Gyimah, Jennifer L. Nicholas, William F. Waters, Carlos Andres Gallegos-Riofrío, Melissa Chapnick, Ivy Blackmore, Katherine E. Douglas, Lora L. Iannotti

**Affiliations:** 1 Brown School, Institute of Public Health, Washington University in St. Louis, St. Louis, MO, USA; 2 Department of Radiology, School of Medicine, Case Western Reserve University, Cleveland, OH, USA; 3 Institute for Research in Health and Nutrition, Universidad San Francisco de Quito, Quito, Ecuador; 4 Gund Institute for Environment, University of Vermont, Burlington, VT, USA; 5 Rollins School of Public Health, Emory University, Atlanta, GA, USA; 6 Department of Pediatrics, Boston Medical Center, Boston, MA, USA

**Keywords:** Ultra-processed foods, Stunting, Bone age, Weight status, Children, NOVA classification, Ecuador

## Abstract

Frequent ultra-processed food (UPF) consumption is consistently associated with poor health outcomes. Little is known about UPF intake during early childhood and its effects on growth. We assessed UPF in relation to child anthropometry, bone maturation, and their nutrition profiles in a rural Ecuadorian community. Covariate-adjusted regression models estimated relationships between UPF intake from a 24-hour Food Frequency Questionnaire and three outcomes: linear growth, weight status and bone maturation. Nutrient Profiling Models (NPM) evaluated a convenience sample of UPF (*n* 28) consumed by children in the community. In this cohort (*n* 125; mean age = 33·92 (sd 1·75) months), 92·8 % consumed some form of UPF the previous day. On average, children consuming UPF four to twelve times per day (highest tertile) had lower height-for-age z-scores than those with none or a single instance of UPF intake (lowest tertile) (*β* = –0·43 [se 0·18]; *P* = 0·02). Adjusted stunting odds were significantly higher in the highest tertile relative to the lowest tertile (OR: 3·07, 95 % CI 1·11, 9·09). Children in the highest tertile had significantly higher bone age z-scores (BAZ) on average compared with the lowest tertile (*β* = 0·58 [se 0·25]; *P* = 0·03). Intake of savoury UPF was negatively associated with weight-for-height z-scores (*β* = –0·30 [se 0·14]; *P* = 0·04) but positively associated with BAZ (*β* = 0·77 [se 0·23]; *P* < 0·001). NPM indicated the availability of unhealthy UPF to children, with excessive amounts of saturated fats, free sugars and sodium. Findings suggest that frequent UPF intake during early childhood may be linked to stunted growth (after controlling for bone age and additional covariates), despite paradoxical associations with bone maturation.

In the Latin American region, many populations continue to undergo nutrition transitions, with increasing amounts of obesogenic foods – including ultra-processed foods (UPF) and beverages – observed in recent dietary patterns^(1,2)^. Overwhelming evidence partially attributes growing trends of obesity globally and in Latin American countries, including Ecuador, to increased consumption of UPF^(1,3–5)^. During the past decade, the coexistence of child under- and overnutrition has been noted in Ecuador^(3,6)^. A recent meta-analysis indicated that nearly 23 % of Ecuadorian children under the age of five had stunted growth, with rural and indigenous children bearing a disproportionate burden of this form of chronic undernutrition^(6)^. Obesity, on the other hand, was prevalent in 8·1 % of children in the same age category^(6)^, marginally exceeding prevalence estimates for child overweight in Latin America (7·5 %) and the world (5·6 %)^(7)^. Recent discourse around the growing double burden of malnutrition continues to acknowledge that many risk factors for obesity and its related outcomes can also be linked to undernutrition outcomes such as stunted growth^(8,9)^. However, frequent UPF intake – a major risk factor for obesity – is rarely discussed in the framing of stunting and other outcomes on the other end of the malnutrition spectrum.

With an increasingly globalised and industrialised food system, sales and consumption of UPF have continued to rise over the past few decades while contributing immensely to population-level energy supplies worldwide^(1,5,10)^. UPF are broadly defined as ready-to-consume, pre-packaged foods that are formulated from a combination of substances extracted from whole foods (e.g. flour, hydrogenated oils and fats, animal derivatives, refined sugars, syrups and starches) and industrial additives or ingredients which are not typically used in culinary preparations within the home environment (e.g. artificial flavours, colours, emulsifiers and preservatives)^(11,12)^. UPF are often characterised by excesses in added sugars, fats and sodium, as well as low levels of dietary fibre. These classic attributes of UPF, along with the extensive industrial processing they undergo, decrease their healthfulness while enhancing their palatability and consequently driving excessive consumption^(11)^. Moreover, compared with unprocessed or minimally processed foods, UPF are often limiting in critical nutrients needed for proper growth and development^(13)^. Yet, worldwide, aggressive marketing and the ubiquitous availability of UPF continue to drive exponential growth in the consumption of these products, often to the detriment of the most vulnerable segments of the population, including children^(14)^.

Dietary patterns that are UPF-dominant have been associated with a myriad of health ramifications across the lifespan^(15,16)^. The effects of excessive UPF consumption during childhood and adolescence, specifically, have also been documented widely in the literature. Comprehensive reviews have reported associations between increased UPF consumption and increased levels of inflammatory biomarkers in children and adolescents^(15)^; increased adiposity in otherwise healthy children^(17)^; increased risk for obesity^(18)^ and poor cardiometabolic health^(19)^. Additionally, evidence links excessive UPF intake with different outcomes that are also associated with stunting, including reduced intake of critical nutrients^(20–22)^ and the disruption of gut microbiome and integrity^(23,24)^. These lines of evidence suggest plausible links between frequent UPF intake and stunted growth in children. There is, however, a paucity of research on UPF consumption during early childhood – particularly, prior to the pre-school phase – and its health implications.

Most of the evidence on UPF consumption has centred on its impact on several indicators of overnutrition, including adiposity, overweight and nutrition-related non-communicable diseases. However, emerging evidence highlights the potential ramifications of increased UPF intake for bone health and linear growth. A recent study in infant rodent models suggested that UPF consumption negatively affects bone ossification, and the researchers indicated that these findings may pose implications for stunted growth in children^(25)^. To our knowledge, this important evidence has not been translated to human populations. Thus, it is imperative that additional research fills this gap to determine whether UPF consumption can qualify as a determinant of growth faltering in young children.

This research has two primary aims and a secondary exploratory aim. First, the study examines the relationship between UPF consumption and child anthropometry (linear growth and weight status). Second, the study examines the relationship between child UPF consumption and bone age, which is a marker of bone maturity and also considered a proxy indicator for and a potential mediator of linear growth^(25,26)^. The secondary aim characterises the nutrition profiles of UPF available to children in the community. We hypothesised that increased UPF consumption will be associated with reduced linear growth, increased weight status and reduced bone age.

## Methods

### Design and study participants

The sample in this study comprises children enrolled in Lulun II, a cohort follow-up study to the Lulun project. The Lulun project was a randomised controlled trial testing the efficacy of incorporating an egg daily into child complementary diets on growth and nutrition outcomes. The trial was conducted in 2015 in the Cotopaxi Province of Ecuador. The cohort was drawn from mixed-indigenous, rural Andean communities dwelling in the following parishes in Cotopaxi: Guaytacama, Mulalo, Pastocalle, Tanicuchi and Toacaso. Details of the Lulun project are described elsewhere^(24)^. In summary, children aged 6–9 months were randomised equally into a control or intervention group, where the family would receive a weekly ration of eggs and provide the index child with an egg daily for a 6-month intervention period. In addition to meeting the age requirement, eligible children had to be in good health with no evidence of egg allergies, fever, a congenital health condition, severe disability nor malnourishment. Children who were multi-birth infants (twins, triplets, etc.) were ineligible to participate in the study. Prior to study enrolment, mothers or primary caregivers went through an informed consent process and provided written consent agreeing to have their child participate in the study. At baseline, caregivers completed surveys on socio-demographic characteristics along with child diet, which was also reported at endline (6 months post-enrolment). Additionally, measures of child anthropometry and blood biomarkers of nutrition were obtained at baseline and endline.

Lulun II was a longitudinal follow-up to assess whether the egg intervention effect on child growth remained after 2 years^(27)^. Mothers and caregivers were contacted and invited to participate in the study. The follow-up study enrolled 135 children out of the 163 children who were originally enrolled in the Lulun project, representing an 83 % retention from the baseline. The sample size for the original randomised controlled trial was based on power calculations estimating an effect size of 0·35 (difference between egg intervention and control groups) after a 6-month intervention period (α = 0·05 and 1 − *β* = 0·90)^(28)^. Although this study was secondary to the original objective to examine whether the growth effect remained 2 years after the egg intervention, we assumed the same effect size and were therefore powered to detect an association at this level. For this analysis, we include children with complete data for all exposure, outcome and covariate variables.

Data collection for Lulun II was conducted 2 years after the end of the original randomised controlled trial, from June to August 2017. Further details on the protocols followed for the Lulun II study can be found elsewhere^(28)^. This study was conducted according to the guidelines laid down in the Declaration of Helsinki, and all study procedures were approved by the ethics committees at Universidad San Francisco de Quito and Washington University in St. Louis. The Lulun and Lulun II projects are registered at https://clinicaltrials.gov under the respective identification numbers: NCT02446873 and NCT03902145.

### Measures

#### Exposures

The primary exposure for this analysis is the frequency of UPF consumption during a 24-hour period. This variable was obtained from responses on child dietary intake from a validated 24-hour Food Frequency Questionnaire (FFQ), which is based on a comprehensive list of foods with their standardised serving/portion sizes identified from extensive formative research^(29)^, our previous studies in young children living in the Cotopaxi region using FFQ^(24)^ and national studies examining child diets using 24-hour recalls in Ecuador^(30)^. Intake frequency was based on different food categories meeting the NOVA classification of UPF specifically adapted for Ecuador’s context^(31,32)^. The NOVA classification establishes four categories: unprocessed or minimally processed; culinary ingredients; processed and ultra-processed^(12)^. For this study, the FFQ included the following UPF categories: juices with added sugars; sodas; sugary drinks; sugary foods (including pre-packaged snacks such as chocolates, sweets, candies, pastries, cakes or biscuits) and salty snacks (pre-packaged savoury snacks). Intake frequencies for these categories were summed to obtain the total frequency of UPF consumed within a 24-hour period for each child. The newly computed variable was subsequently divided into tertile ranks based on the number of times that UPF were consumed during the past day. Tertiles were selected instead of quartiles and quintiles in view of the small sample size and need for sufficient number of cases in each category to run analyses. Tertile ranks were created using the *n*-tile function in R software (where *n* 3), which equally distributes cases in the sample according to the spread/range in frequency of UPF intake within the cohort (0–12 times). Although specific portion sizes are not captured here, ranking intake of specific food groups using FFQ is a recommended practice for dietary assessments^(33)^. In fact, others have applied FFQ rankings of UPF intake, without specific portions captured, to estimate the risk of mortality in a large representative sample^(34)^.

To explore potential differential effects from different categories of UPF on the outcomes of interest, we created three binary categorical variables capturing any consumption in the previous 24 hours: *salty snacks*; *sugary foods* and *sugar-sweetened beverages (SSB)* (encompassing juices with added sugars, sodas and sugary drinks).

#### Outcomes

For our primary aims, the outcomes examined were child anthropometry (height-for-age z-score (HAZ), weight-for-height z-score (WHZ)) and bone maturity (bone age z-score (BAZ)). For the anthropometric outcomes, measurements were conducted following the WHO’s guidelines on assessing child growth^(35)^. Child heights were measured by two enumerators using a stadiometer (Seca GmbH & Co KG). Heights were measured twice, and if a difference of 5 mm was observed between the first and second measures, a third height measure was obtained. Weights were measured twice using a Seca Model 874 Electronic Digital Scale (Seca GmbH & Co KG), and a third measure was obtained if there was a difference of 0·05 kg between the first two measures.

Heights and weights were converted to HAZ and WHZ scores, respectively, according to the WHO Growth Standards^(35)^. The WHZ is used instead of the weight-for-age z-score (WAZ) for this analysis because WHZ is the measure used to indicate overweight in children under the age of five^(36)^. HAZ scores were also used to create a binary categorical variable indicating stunting status (HAZ < –2).

Bone age was measured using tablet-based ultrasound methods capturing images of the hand and wrist. Bone age represents bone maturation or advancement and is defined by the degree of ossification of the carpal bones and epiphyses of the metacarpals and phalanges of the fingers (online Supplementary Fig. 1)^(26,37,38)^. During early childhood, ossification centres in the hand and wrist are used to evaluate skeletal maturity^(37)^.The methodology for evaluating bone age in this study is detailed elsewhere^(26)^. In brief, a Lumify L12–4 broadband linear array transducer (Philips Healthcare) was connected to a Samsung S2 Galaxy tablet (Samsung Electronics) and used to obtain targeted images of different ossification centres located in the hand and wrist. Specific bones captured are detailed in online Supplementary Table 1. This table also details bones which are measured with adjustments made for child sex and age, according to the standards of Greulich and Pyle^(39)^. These standards recognise sex-mediated differences in the maturation rate of these specific bones. Bone ages were evaluated twice at two distinct periods by a board-certified radiologist who was an investigator on the Lulun project, and intra-rater reliability was assessed. Bone ages were subsequently converted to z-scores (BAZ)^(26)^. For additional analyses, we computed a binary variable to indicate low bone age (BAZ < –2); this definition and cut-off have been applied previously in other paediatric studies using the standards of Greulich and Pyle to assess bone age^(26,40,41)^.

#### Covariates

The first set of covariates included in the analyses were child characteristics – child age as a continuous variable and child sex as a binary categorical variable. Additional covariates selected for this analysis were identified as confounders which influence both the exposure and outcome. These include proxies of household/caregiver socio-economic status: caregiver employment status, involvement in food production and livestock ownership. All three socio-economic variables are binary categorical variables. We also account for the child’s group assignment from the original Lulun trial (egg intervention *v*. control) to control for any potential intervention effects that may have resulted from the trial. Nevertheless, in this cohort, we found that the intervention effects on growth that were observed at the end of the randomised controlled trial were no longer present 2 years later^(28)^. Adjusting for energy intake is a common practice in nutritional epidemiology. For this analysis, we use dietary diversity as a proxy indicator, given evidence shows that it can adequately substitute measurements for predicted energy intake ratios among different populations^(42)^. Here, the variable describes whether or not the child met the recommended minimum dietary diversity (MDD), which is determined by the consumption of foods from at least four out of a possible seven food groups embedded in the WHO’s MDD indicator^(43)^.

### Statistical analysis

#### Descriptive statistics

First, descriptive statistics were used to profile the study population according to child demographic characteristics, morbidities, anthropometry and nutrition. Additionally, household socio-economic status is detailed at this stage of analysis. Analyses with the one-way ANOVA tested differences for continuous variables, and the Pearson’s *χ* ^2^ test assessed statistical differences for categorical variables. When contingency tables for categorical variables had cells with fewer than five cases, the Fisher’s exact test was used to evaluate those factors.

The second phase of the descriptive statistics involved analyses determining differences in any consumption of different food categories during the past 24 h, according to the tertile ranks of UPF consumption. Foods within these categories are within the NOVA classification of whole unprocessed or minimally processed foods, within Ecuador’s context^(31,32)^. Distinct food categories selected from the 24-h FFQ for these analyses were fish and other seafood; eggs; dairy products; legumes; cereals and grains; and fruits and vegetables. Assessments of the MDD score as well as the three specific groups of UPF are also included in this phase. The Kruskal–Wallis test assessed differences in MDD scores across the ranks of UPF consumption.

#### Regression modelling

Linear regression models estimated the respective relationships between the frequency of UPF consumption and the continuous outcomes of interest: HAZ, WHZ and BAZ. For all three continuous outcomes, covariates were introduced into the models drawing on the evidence base for their potential role as confounding factors. For example, socio-economic factors may influence both UPF consumption patterns and growth and bone maturation parameters. For HAZ, the model includes BAZ in addition to the covariates, given previous evidence within this same cohort that showed significant correlations between BAZ and HAZ^(26)^. The inclusion of this variable also recognises bone age as a potential mediator in the pathway for the hypothesised relationship between UPF consumption and linear growth^(25)^. Influential cases were identified in models after computing their Cook’s distances and visualising them on a Cook’s distance plot. New models were created without those influential cases. Subsequently, we compared effect sizes for the two models to determine whether the influential points affected the estimates. After modelling, we assessed the quality of each model using evaluations of multicollinearity represented by variation inflation factors, linearity between predicted values and residuals, homogeneity of variance and normality of residuals.

Logistic regression models were used for further analyses to estimate the odds of child stunting and low bone age in relation to UPF intake. We considered developing logistic regression models for a weight-related outcome (overweight – WHZ > 2); however, there were no children in the sample who met the overweight criteria. Logistic regression models were similar to those described for the linear models, with BAZ incorporated into the model for stunting status. The impact of influential cases on models was assessed using Cook’s distance values. Box–Tidwell transformations assessed the linearity assumption for continuous independent variables. Multicollinearity was also examined via variation inflation factor values.

#### Mediation analysis

**Fig. 1.**
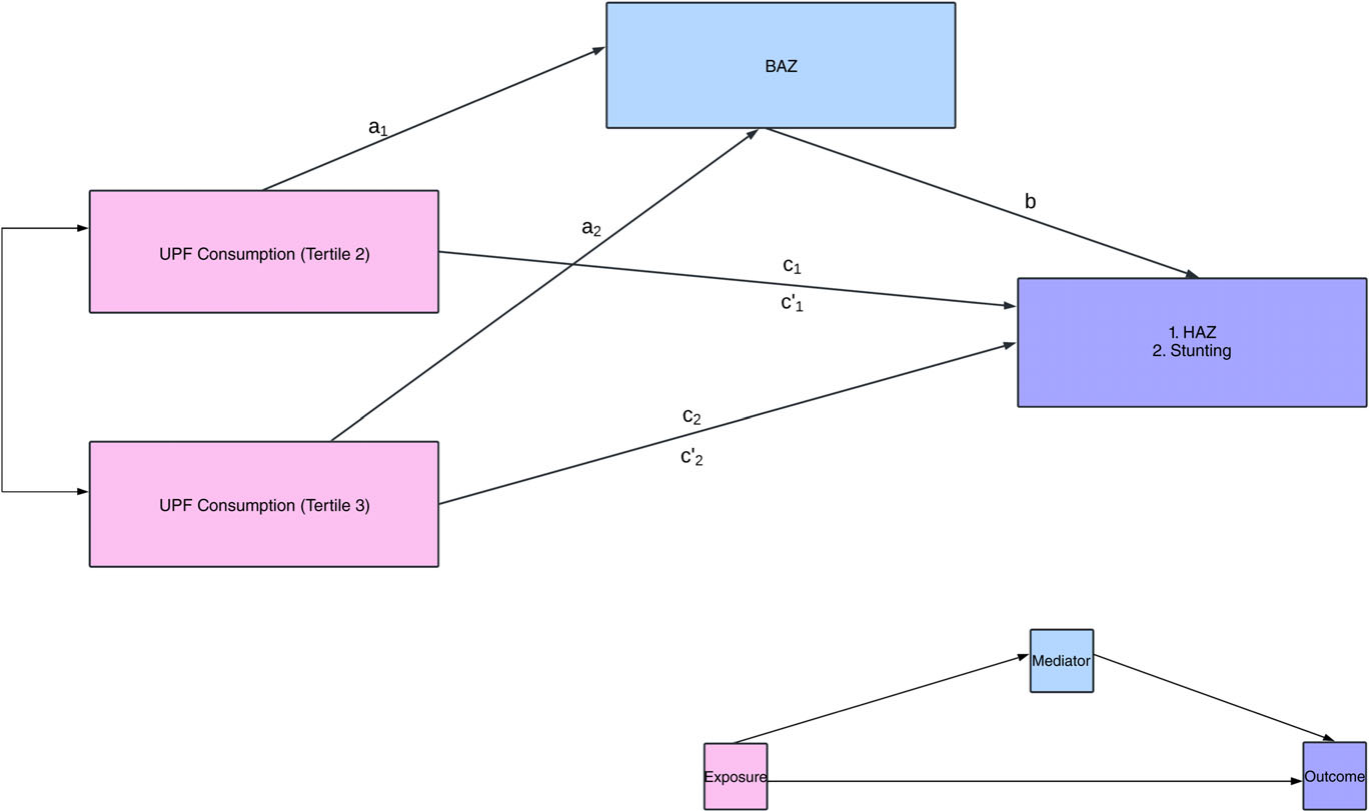
Mediation model: total effect: effect of UPF consumption (tertile 2 (*c*_*1*_) and tertile 3 (*c*_*2*_)) on HAZ or stunting. Direct effect: effect of UPF consumption (tertile 2 (*c'*_*1*_) and tertile 3 (*c'*_*2*_)) on HAZ or stunting, after controlling for BAZ. Indirect effect: effect of UPF consumption (tertile 2 (*a*_*1*_ *× b*) and tertile 3 (*a*_*2*_ *× b*) on HAZ or stunting, mediated by BAZ. tertile 1: consumption of UPF 0–1 times per day (*reference group, not shown in model)*; tertile 2: consumption of UPF 2–3 times per day; tertile 3: consumption of UPF 4–12 times per day. UPF, ultra-processed food; HAZ, height-for-age z-score; BAZ, bone age z-scores.

Using the R package *medflex* ^(44)^, we tested BAZ as a mediating factor in the relationships between UPF consumption and the respective indicators for linear growth: HAZ and stunting status. We also control for confounders used in the regression modelling between UPF consumption and HAZ/stunting during this analysis. This analysis was warranted in recognition of emerging evidence on the relationship between UPF consumption and parameters of bone quality^(25)^ as well as previous findings from this study cohort correlating bone age with linear growth^(26)^. Using natural effects models, the analysis estimates the natural direct and indirect effects for each tertile of UPF intake, along with 95 % CI generated via robust bootstrapping procedures. Wald *χ* ^2^ tests through analysis of deviance was subsequently used for an overall assessment of the mediation model to determine whether the natural indirect effects for the individual tertiles of UPF consumption differ. Figure 1 provides a detailed visual of the hypothesised mediation analysis model.

All analyses were conducted in R software (version 3.6.2).

### Nutritional profiling of ultra-processed foods

Nutritional profiling methods are used to assess the degree of healthfulness of foods and can be used to draw conclusions on a food environment of interest. For this secondary aim, we obtained UPF products and wrappers specifically in and around a primary school located in the parish of Pastocalle, located in the Cotopaxi Province in the central Ecuadorian highlands. Although the items sampled were collected in a school setting, they may be representative of the kinds of UPF available to young children in the community of interest. We obtained a convenience sample of snack and candy wrappers littered on the ground and purchased candy and packaged snacks sold in the school cafeteria and immediately outside the school gate. Prior to analysis, we examined the sample of wrappers and packages to ensure that they met the criteria for UPF according to the NOVA classification. We approach the profiling of the sampled food items using two widely used models: respective Nutrient Profiling Models (NPM) established by the UK’s Department of Health^(45)^ and the Pan American Health Organization (PAHO)^(46)^. Henceforth, we refer to the respective models as UK NPM and PAHO NPM.

The PAHO NPM sets criteria for the concentration of sodium; energy intake from free sugars, saturated fat, trans fat; and the presence of artificial sweeteners in order to assess the content of UPF by identifying those containing excessive amounts of these components. For this analysis, we concentrated on the established limits for sodium, free sugars and saturated fats in order to be consistent with the specific nutrients or dietary indicators used in the UK NPM assessments. For each product, free sugars were determined by the value provided for one of the following: declared added sugars, declared total sugars when there are little to no ingredients with naturally occurring sugars or 50 % of declared total sugars for dairy products (e.g. sweetened milk or yogurt)^(46)^. Excessive amounts of sodium, saturated fats and free sugars are defined by thresholds detailed in Table 1 ^(2)^.

**Table 1.** Criteria for identifying ultra-processed foods containing excessive sodium, saturated fats, and free sugars, according to the Pan American Health Organization^(2)^

Nutrient	Considered excessive when food item contains:	Energy determination
Sodium	≥ 1 mg of sodium per 1 kcal	N/A
Saturated fats	≥ 10 % of total energy from saturated fat	Amount of saturated fats (g) × 9
Free sugars	≥ 10 % of total energy from free sugars	Amount of free sugars (g) × 4

There has been some debate in the nutrition research community regarding approaches to evaluating UPF, where energy density, nutrition facts and levels of processing are often the only factors considered without accounting for other potential dimensions of nutritional value – for example, nutrient fortification and the inclusion of whole grains^(47–49)^. The UK NPM allows consumers and researchers to view food items more holistically by evaluating UPF beyond specific nutrients, while accounting for ingredients and factors such as whole fruits, nuts, vegetables, fibre and protein content which could contribute to the healthfulness of UPF. Although it was originally designed for the British context, the model has been used for UPF and food environment evaluations in other places in the world^(50)^, including Latin America^(51,52)^.

Full details on the methodology can be found elsewhere^(45)^. Briefly, the UK NPM scores foods or drinks in three stages. The first stage scores the food item accounting for total energy in kJ, saturated fats in g, sugar in g and Na in mg per 100 g of each food item. The second stage results in a separate score based on the percentage of whole fruits, nuts and vegetables (as a single category); fibre in g and protein in g per 100 g of each food item. In this second stage, protein can only be accounted for if a food item that scores less than 11 points at the first stage or has 5 or more points for the percentage of fruit, vegetables and nuts. In the third stage, scores from the second stage are subtracted from the first to obtain a final UK NPM score. Beverages scoring 1 point or more are categorised as less healthy, whereas foods are less healthy if they have a score of 4 or greater.

For the present study, a survey with formulas built into a branching logic was developed within the Research Electronic Data Capture (REDCap) software^(53)^ to calculate the UK NPM scores for each food item. Using the labels on each package or wrapper, for each food item in our sample, we recorded the data per 100 g for all the nutrition information required for both NPM as previously described. In cases where nutrition information was not available, we searched the USDA Food Composition Database for information on items either produced by the same company or generics. For instance, for a marshmallow snack with incomplete nutrition information, we source the data for a generic marshmallow product in the USDA Food Composition Database. For the PAHO NPM, we calculated the energy obtained from free sugars and saturated fats following PAHO criteria described in Table 1. These calculations, along with data on sodium content, were used to determine whether each food item contained excessive free sugars, saturated fats or sodium according to the energy-related criteria set by PAHO (Table 1).

## Results

### Sample characteristics

For the study’s primary aims, the analysis accounts for a sample of 125 children from Lulun II with complete data. The median frequency of UPF consumption was three times per day (interquartile range: 1–5 times per day), and 92·8 % of the children (*n* 116) consumed some form of UPF the previous day. Univariate analyses detected a statistically non-significant relationship between stunting and frequency of UPF consumption, although the prevalence of stunted growth for children in the highest tertile of UPF consumption was comparatively higher (61·0 %) than among children in the lowest tertile (47·6 %) (*P* = 0·17). Differences in mean HAZ scores were also statistically non-significant. Univariate analyses showed a non-significant association between weight parameters and frequency of UPF consumption (*P* = 0·79). However, WHZ scores were lowest for children in the highest tertile of UPF consumption (0·45 [sd 0·73]). Mean BAZ scores were significantly different across the tertiles of UPF consumption, with children in the highest tertile having higher BAZ scores on average (–0·94 [sd 1·27]) compared with children in the other tertiles (*P* = 0·02).

Statistically non-significant differences were observed across the tertiles of UPF consumption for all other sample characteristics explored in the univariate analyses, as detailed in Table 2. Generally, the occurrence of co-morbidities – fever and diarrhoea – from 2-week morbidity recalls, as reported by caregivers, was highest for children in the highest tertile of UPF consumption.

**Table 2.** Child characteristics by frequency of UPF consumption (times per day) *n* 125 (Mean values and standard deviations)

	Tertile of UPF consumption								
	Full sample		Tertile 1 (*n* 42)		Tertile 2 (*n* 42)		Tertile 3 (*n* 41)		
UPF intake (times per day), range	0—12		0—1		2—3		4—12		
	Mean or %	sd	Mean or %	sd	Mean or %	sd	Mean or %	sd	*P*
Child characteristics									
Child age (months)	33·92	1·75	34·28	1·68	33·77	1·69	33·71	1·87	0·27‡
Child sex (female)	45·6 %	–	33·3 %	–	54·8 %	–	48·8 %	–	0·13‡
Child health and morbidities									
Any vitamin/mineral supplementation, last 6 months	36·8 %	–	33·3 %	–	42·9 %		34·1 %	–	0·61‡
Any diarrhoea, past 2 weeks	21·6 %	–	16·7 %	–	21·4 %		26·8 %	–	0·53‡
Any bloody stool, past 2 weeks	3·2 %	–	4·8 %	–	4·8 %		0·0 %	–	0·54§
Any fever, past 2 weeks	20·8 %	–	19·0 %	–	19·0 %		24·4 %	–	0·79‡
Household (caregiver) socio-economic status									
Currently employed	50·4 %	–	52·4 %	–	47·6 %		51·2 %	–	0·90‡
Livestock ownership	95·2 %	–	90·5 %	–	100·0 %		95·1 %	–	0·12§
Involvement in food production	72·0 %	–	66·7 %	–	83·3 %		65·9 %	–	0·13‡
Anthropometric and bone measures									
HAZ	–2·06	0·90	–2·04	0·86	–1·88	0·85	–2·27	0·95	0·14†
Stunting (HAZ < −2)	49·6 %	–	47·6 %	–	40·5 %		61·0 %	–	0·17‡
WHZ	0·51	0·74	0·55	0·77	0·54	0·74	0·45	0·73	0·79†
BAZ	–1·20	1·17	–1·58	1·15	–1·06	0·98	–0·94	1·27	0·02*,†
Low bone age (BAZ < −2)	24·8 %	–	35·7 %	–	16·7 %		22 %	–	0·11‡

UPF, ultra-processed food; HAZ, height-for-age z-score; WHZ, weight-for-height z-score; BAZ, bone age z-score.

*P values indicate statistical significance (*P* < 0·05).

† One-way ANOVA.

‡ Pearson’s *χ* ^2^ test.

§ Fisher’s exact test.

### Consumption patterns

Table 3 details the consumption patterns in the cohort. Besides the consumption of cereals and grains, differences in the consumption patterns for whole or minimally processed food groups were not statistically significant across the tertile ranks. Nearly all the children sampled consumed cereals and grains during the previous day (96·8 %), and all children in the second and third tertiles consumed cereals and grains (100 %). In addition, nearly all the children in the sample consumed some fruit and vegetable, regardless of the tertile rank of UPF consumption. Assessments of the MDD scores showed high dietary diversity across each tertile of UPF consumption, with most children meeting the MDD requirement of consuming foods from four or more groups.

**Table 3.** Consumption patterns by frequency of UPF consumption (times per day) *n* 125 (Mean values and standard deviations)

	Tertile of UPF consumption								
	Full sample		Tertile 1 (*n* 42)		Tertile 2 (*n* 42)		Tertile 3 (*n* 41)		
UPF intake (times per day), range	0—12		0—1		2—3		4—12		
	Mean or %	sd	Mean or %	sd	Mean or %	sd	Mean or %	sd	*P*
Dietary diversity									
MDD score	4·76	1·17	4·76	1·19	4·81	1·31	4·71	1·03	0·96†
Met minimum dietary diversity	83·2 %		83·3 %		83·3 %		82·9 %		0·99‡
Any consumption, previous day									
Fish and other seafoods	14·4 %		16·7 %		7·1 %		19·5 %		0·24‡
Eggs	64·0 %		71·4 %		59·5 %		61·0 %		0·46‡
Dairy products	72·0 %		76·2 %		66·7 %		73·2 %		0·61‡
Legumes	24·8 %		26·2 %		28·6 %		19·5 %		0·61‡
Cereals and grains	96·8 %		90·5 %		100·0 %		100·0 %		0·03*
Fruits and vegetables	99·2 %		100·0 %		97·6 %		100·0 %		1·00§

UPF, ultra-processed food; MDD, minimum dietary diversity.

*P values indicate statistical significance (*P* < 0·05).

† Kruskal–Wallis test.

‡ Pearson’s *χ* ^2^ test.

§ Fisher’s exact test.

**Fig. 2.**
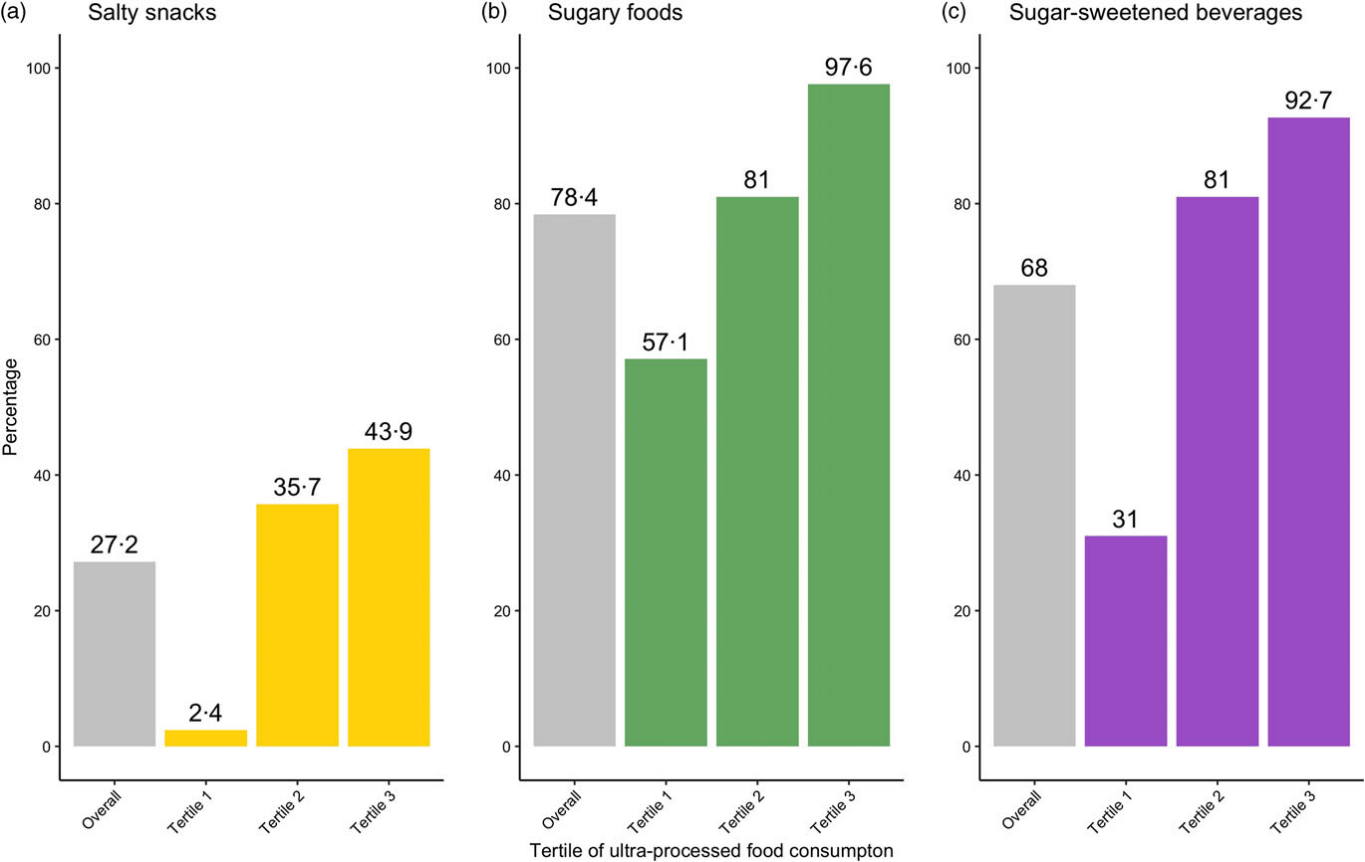
Consumption prevalence for ultra-processed food groups: bars in grey on the farthest left of each ultra-processed food category (a) salty snacks; (b) sugary foods; (c) sugar-sweetened beverages represent the proportion of children within the cohort studied (*Overall*) (*n* 125) who consumed food from a given group the day prior. The three additional bars represent the proportion of children within each tertile (*Tertile 1* (*n* 42); *Tertile 2* (*n* 42)*, Tertile 3* (*n* 41)) who consumed food from a given group the day prior.

Analyses of differences for the three categories of UPF consumption showed statistical significance across all tertiles of UPF intake (*P* < 0·001), with more than 90 % of children in the highest tertile consuming some form of SSB or sugary food/snack the previous day. Figure 2 shows consumption frequencies observed for the overall sample and for each tertile within each category of UPF. SSB were consumed by 92·7 % of children in the highest tertile, compared with 31·0 % of children in the lowest tertile. Sugary food/snack consumption was observed in almost all children in the highest tertile (97·6 %), compared with more than half of children in the lowest tertile (57·6 %). The salty snack category was the least consumed among all the three UPF groups.

### Associations between ultra-processed food consumption and growth parameters

#### Linear growth and stunting

After controlling for confounding variables, dietary diversity as a proxy for energy intake and child bone age, the regression model for HAZ showed a statistically significant negative magnitude of association between the frequency of UPF consumption and HAZ for the highest tertile of UPF consumption: on average, children in the highest tertile of UPF consumption had HAZ scores that were 0·43 units lower compared with children in the lowest tertile (*β* = –0·43 [se 0·18]; *P* = 0·02) (Table 4). Statistically non-significant associations were detected for the relationship between any consumption within the individual UPF groups and HAZ; however, all the associations were negative (Table 5).

**Table 4.** Association between the frequency of UPF consumption and growth indicators – HAZ, WHZ and BAZ (*β*-coefficients and standard errors)

	Tertile 1			Tertile 2			Tertile 3		
	*β*-coefficient	se	*P*	*β*-coefficient	se	*P*	*β*-coefficient	se	*P*
HAZ†	Reference			0·01	0·19	0·97	–0·43	0·18	0·02*
WHZ‡	Reference			0·03	0·17	0·87	–0·09	0·16	0·56
BAZ‡	Reference			0·46	0·26	0·08	0·58	0·25	0·03*

UPF, ultra-processed food; HAZ, height-for-age z-score; WHZ, weight-for-height z-score; BAZ, bone age z-score.

* *P* values indicate statistical significance for *β*-coefficients (*P* < 0·05).

† Model is adjusted for child BAZ, child sex; child age; parental/caregiver socio-economic characteristics: employment status, involvement in food production, livestock ownership; meeting minimum dietary diversity and group assignment from the Lulun project.

‡ Model is adjusted for child sex; child age; parental/caregiver socio-economic characteristics: employment status, involvement in food production, livestock ownership; meeting minimum dietary diversity and group assignment from the Lulun project.

**Table 5.** Association between consumption of specific UPF groups and growth indicators – HAZ, WHZ and BAZ (*β*-coefficients and standard errors)

	No			Yes		
	*β*-coefficient	se	*P*	*β*-coefficient	se	*P*
Salty snacks						
HAZ†	Reference			–0·06	0·18	0·75
WHZ‡	Reference			–0·30	0·14	0·04*
BAZ‡	Reference			0·77	0·23	< 0·001*
Sugary foods						
HAZ†	Reference			–0·24	0·18	0·20
WHZ‡	Reference			–0·26	0·16	0·10
BAZ‡	Reference			0·53	0·25	0·04*
Sugar-sweetened beverages						
HAZ†	Reference			–0·07	0·16	0·67
WHZ‡	Reference			0·04	0·14	0·76
BAZ‡	Reference			0·13	0·23	0·58

UPF, ultra-processed food; HAZ, height-for-age z-score; WHZ, weight-for-height z-score; BAZ, bone age z-score.

* *P* values indicate statistical significance for *β*-coefficients (*P* < 0·05).

† Model is adjusted for child BAZ, child sex; child age; parental/caregiver socio-economic characteristics: employment status, involvement in food production, livestock ownership; meeting minimum dietary diversity and group assignment from the Lulun project.

‡ Model is adjusted for child sex; child age; parental/caregiver socio-economic characteristics: employment status, involvement in food production, livestock ownership; meeting minimum dietary diversity and group assignment from the Lulun project.

**Fig. 3.**
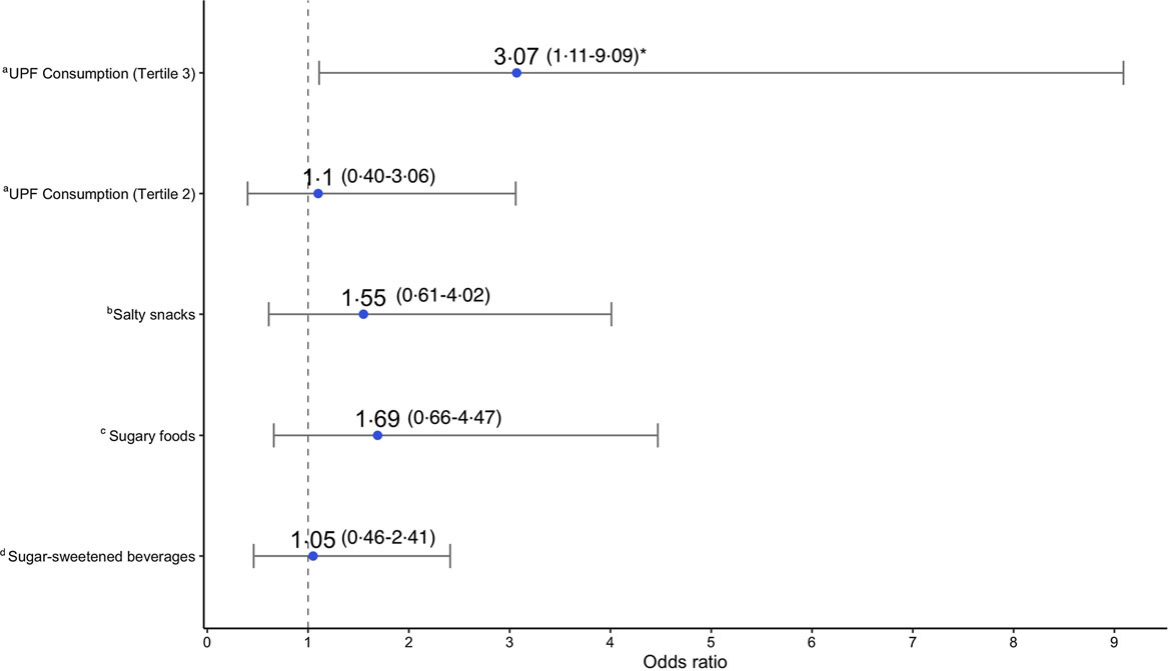
Association between ultra-processed food consumption and stunting (HAZ < –2): adjusted OR and 95 % CI showing the association between different UPF exposures and stunting (HAZ < –2), determined by logistic regression models. Each model was adjusted for child BAZ, child sex; child age; parental/caregiver socio-economic characteristics: employment status, involvement in food production, livestock ownership; meeting minimum dietary diversity, and group assignment from the Lulun project. ^a^Model where *frequency of UPF consumption according to tertiles* is the primary exposure; reference group – tertile 1(consumption 0–1 time a day). ^b^Model where *any salty snack consumption* is the primary exposure; reference group – no salty snack consumption. ^c^Model where *any sugary food consumption* is the primary exposure; reference group – no sugary food consumption. ^d^Model where *any sugar-sweetened beverage consumption* is the primary exposure; reference group – no sugar-sweetened beverage consumption. *indicates statistical significance (*P* < 0·05). HAZ, height-for-age z-score; UPF, ultra-processed food; BAZ, bone age z-score.

Adjusted logistic regression models for stunting detected a significant relationship with UPF consumption frequency for the highest tertile (Fig. 3). Here, children in the highest tertile had nearly three times higher odds of stunting compared with children in the lowest tertile (OR: 3·07, 95 % CI 1·11, 9·09). Respective models for the three individual categories of UPF showed non-significant relationships with stunting, although stunting odds were generally higher for children who had consumed any SSB, sugary snacks or savoury snack – ranging from 5 % to 69 % higher odds (Fig. 3).

#### Weight status

Apart from salty snacks, all other UPF exposures showed statistically non-significant relationships with the weight outcome (WHZ). Consumption of salty snacks correlated negatively with child WHZ (*β* = –0·30 [se 0·14]; *P* = 0·04). Negative, but non-significant, magnitudes of association were detected for the highest tertile of UPF consumption (*β* = –0·09 [se 0·16]; *P* = 0·56) and sugary snacks/foods (*β* = –0·26 [se 0·16]; *P* = 0·10) (Tables 4 and 5).

#### Bone age

**Fig. 4.**
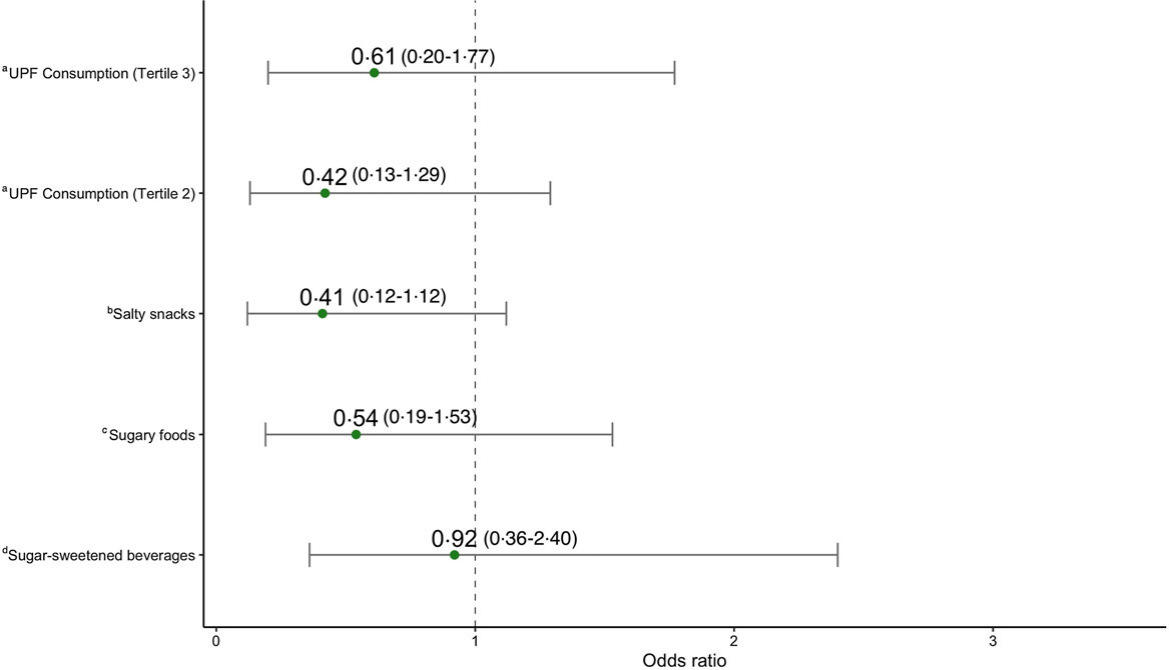
Association between ultra-processed food consumption and low bone age (BAZ < –2): adjusted OR and 95 % CI showing the association between different UPF exposures and low bone age (BAZ < –2), determined by logistic regression models. Each model was adjusted for child sex; child age; parental/caregiver socio-economic characteristics: employment status, involvement in food production, livestock ownership; meeting minimum dietary diversity, and group assignment from the Lulun project. ^a^Model where *frequency of UPF consumption according to tertiles* is the primary exposure; reference group – tertile 1(consumption 0–1 time a day). ^b^Model where *any salty snack consumption* is the primary exposure; reference group – no salty snack consumption. ^c^Model where *any sugary food consumption* is the primary exposure; reference group – no sugary food consumption. ^d^Model where *any sugar-sweetened beverage consumption* is the primary exposure; reference group – no sugar-sweetened beverage consumption. BAZ, bone age z-score; UPF, ultra-processed food.

The adjusted model for BAZ showed a statistically significant and positive association between frequency of UPF consumption and BAZ for children in the highest tertile (Table 4). Children in the highest tertile of UPF consumption had BAZ scores that were 0·58 units higher on average, relative to children in the lowest tertile (*β* = 0·58 [se 0·25]; *P* = 0·03). Respective models for the three categories of UPF were all positively associated with BAZ. Statistical significance was however detected only for consumption of salty snacks (*β* = 0·77 [se 0·23]; *P* < 0·001) and sugary foods/snacks (*β* = 0·53 [se 0·25]; *P* = 0·04) (Table 5). Adjusted regression models estimating the odds of low bone age were all statistically non-significant but showed negative magnitudes of association (Fig. 4).

#### Bone age-mediated relationships between ultra-processed food consumption and indicators of linear growth

Indirect effects were not statistically significant for both HAZ scores and stunting, demonstrating limited evidence for bone age as a mediator (online Supplementary Table 2). However, a statistically significant relative direct effect was observed between the highest tertile of UPF consumption and stunting (effect size = 1·07, bootstrap 95 % CI 0·06, 2·07) (online Supplementary Table 2). This significant direct effect parallels findings reported for the adjusted odds of stunting for the highest UPF consumption tertile.

### Nutrition profiling

For the study’s secondary aim, we assessed twenty-eight items with adequate data for nutrition profiling analyses. There were no SSB in the sample, while 78·6 % (*n* 22) of the sample consisted of sugary snacks, and the remaining were salty/savoury snacks. The mean UK NPM score for the sample was 14·89 (sd 7·58), indicating that UPF found in the school environment were unhealthy; scores of 4 and greater are considered ‘less healthy’^(45)^. The mean UK NPM score for the salty snacks was 16·59 (sd 7·50), whereas sugary snacks scored 14·45 (sd 7·72) on average. Only one food item was considered ‘healthy’ according to the NPM scoring. However, this item was entirely composed of industrially formulated ingredients (product ID 12 in Table 6).

**Table 6.** List of UPF sampled and summary of individual nutrition profiles*

ID	Product name	Energy, kcal	Saturated fat, g	Energy from saturated fats, kcal	Excess saturated fats	Free sugars, g	Energy from free sugars, kcal	Excess free sugars	Na, mg	Excess Na	Overall UK NPM score
1	Sorbetitos Sami†	401·52	0	0	No	100	400	Yes	0	No	15
2	Doritos Lemon Remix	501·11	9·99	89·91	Yes	0	0	No	765·9	Yes	23
3	Gelactica (Gomitas de Frutas/Jelly Fruit)†	100·38	0	0	No	25	100	Yes	0	No	6
4	Recontra Acido boca loca (Red)†	200·76	0	0	No	20	80	Yes	0	No	6
5	Sabor Uva Lengua†	301·14	0	0	No	75·3	301·2	Yes	20	No	13
6	Chicle perlitas acido†	301·14	0	0	No	80	320	Yes	0	No	13
7	Paletaufo Krazymon Maracuyá con Sal y Lemon†	301·14	0	0	No	50	200	Yes	10 600	Yes	23
8	Papa Nic Maracuya	412·54	0	0	No	0	0	No	2735·2	Yes	10
9	Papa Nic Limon	350·14	0	0	No	0	0	No	2700	Yes	9
10	Recontra Acido boca loca (yellow)†	200·76	0	0	No	20	80	Yes	0	No	6
11	Chicle perlitas†	301·14	0	0	No	80	320	Yes	0	No	13
12	Gelatina Sabor a frutas†	5·81	0	0	No	0	0	No	0	No	0
13	Paletaufo Krazymon Mango con Sal y Lemon†	301·14	0	0	No	50	200	Yes	10 600	Yes	23
14	Los Habiteñitos Habitas	599·89	10	90	Yes	0	0	No	550	No	24
15	Nucita (Crema con sabor a leche, chocolate y nueces)†	510·98	4·44	39·96	No	60	240	Yes	122	No	21
16	Salticas Galletas Salatidas	500·61	8·33	74·97	Yes	6·66	26·64	No	799	Yes	23
17	TaniLact†	97·54	1·59	14·31	Yes	8·48	33·92	No	42·4	No	4
18	Colombina Piazza (barquillos Sabor a malteada de fresa)†	503·98	11·6	104·4	Yes	38·8	155·2	Yes	78	No	20
19	Circus Galletas con crema sabor a fresa†	465·02	8·93	80·37	Yes	32·1	128·4	Yes	410·6	No	24
20	Topsy choc ice cream†	119·62	4·59	41·31	Yes	8·25	33	Yes	12·87	No	6
21	Yellies Marshmallows (helados rellemos)†	301·14	0	0	No	60	240	Yes	0	No	13
22	Tango Báilate (Sabor a chocolate)†	521·02	12	108	No	44	176	Yes	160	No	25
23	Pipa Chos	651·28	5	45	No	0	0	No	250	No	10
24	Led Chupi Tops†	334·60	0	0	No	62·5	250	Yes	37·5	No	14
25	Golpe Oblea Rellena Cubrieta con Caramelo y Cereal Crocante†	519·08	11·1	99·9	Yes	59·2	236·8	Yes	111	No	24
26	Orbita Galettas Rellenas con Crema Sabor a vanilla con Cobertura sabor a chocolate†	480·87	16	144	Yes	36	144	Yes	120	No	25
27	Frunas Frutas†	84·61	2·08	18·72	Yes	56·2	224·8	Yes	0	No	13
28	Colombina Max (Frutacidas)†	84·37	0	0	No	64·7	258·8	Yes	59	No	11

UPF, ultra-processed food, NPM, Nutrient Profiling Models.

* All quantities are per 100 g of product sample.

† Indicates sugary/sweetened snack.

Following criteria described for the PAHO NPM in Table 1, we found that 71·4 % of the UPF sampled had excessive free sugars, 21·4 % had excessive Na and 35·7 % had excessive saturated fats. Table 6 lists the food items sampled along with their individual UK NPM scores as well as their characteristics according to the PAHO NPM criteria. More detailed nutrition data used in the profiling assessment can be found in online Supplementary Table 3.

## Discussion

This study assessed UPF consumption in relation to child anthropometry and bone maturation in children who participated in the Lulun II study. We also assessed the nutrition profiles of UPF found in and around a primary school, thereby allowing us to characterise specific UPF in the community that may influence the child outcomes studied. Although the Lulun project demonstrated significant effects of an egg intervention on preventing stunted growth^(24)^, findings from the Lulun II follow-up indicated that this intervention effect was no longer present 2 years later^(28)^. Further research was therefore needed to understand determinants of growth faltering – among the myriad of factors associated with stunting – in this cohort. Here, we assessed UPF consumption as one potential driving factor for stunted growth. Findings from this study suggest that increased consumption of UPF may be associated with increased bone maturation, while increasing the risk of stunted growth or chronic malnutrition in young children. The latter outcome is particularly compelling, because stunted infants or young children generally cannot recover and may bear the risks of long-term ramifications on health and productivity throughout the life course^(54)^. Our findings also showed limited evidence for UPF impacts on weight status and for bone maturation as a mediator in the relationship between UPF intake and the indicators of linear growth. Nutrition profiling analyses provided evidence for a potentially unhealthy food environment in a school setting, based on the kinds of products consumed by primary schoolchildren.

Our findings suggest that frequent UPF intake is negatively associated with linear growth. There is limited evidence directly associating UPF consumption with stunted growth. One study by Pries and colleagues^(22)^ with a cohort from Nepal found a significant relationship between higher total energy intake from complementary ultra-processed snack foods and lower length-for-age z-scores. The researchers also found associations with increased UPF intake and dietary inadequacy, which also partially mediated the relationship between high UPF intake and low length-for-age z-scores^(22)^. Emerging literature supports the evidence from Nepal suggesting nutrient inadequacy as a potential pathway explaining the relationship between UPF intake and decreased linear growth.

First, diets characterised by high UPF consumption may signify that UPF are displacing nutrient-dense unprocessed or minimally processed foods in the traditional Ecuadorian diet^(3,55,56)^. A population-based survey of Canadians aged 2 years and above found that UPF consumed by the population, compared with unprocessed or minimally processed foods in the diet, had lower mean nutrient concentrations for proteins, fibre, minerals (Zn, Fe, Mg, Ca, P and K) and vitamins (A, C, D, B_12_ and other critical B vitamins)^(13)^. Similarly, in a representative longitudinal analysis of US adults, individuals in the highest quartile of UPF consumption had significantly lower scores for the Nutrient-Rich Foods Index and the Healthy Eating Index^(34)^, two indicators for nutritional quality in one’s diet. During early childhood, poor dietary quality is associated with sub-optimal growth outcomes^(57)^.

Displacement of nutrient-dense foods in the diet may affect the intake of nutrients for which deficiencies are linked to stunting pathogenesis. In a representative sample of Chilean pre-schoolers, researchers found that children with UPF-dominant diets had significantly reduced intake of proteins, polyunsaturated fatty acids (PUFA), Zinc, folate, fibre and vitamin A^(21)^. Similarly, a study of pre-school children in Brazil found negative associations between high UPF consumption and intake of key nutrients, including proteins and fibre, as well as consumption of unprocessed or minimally processed foods^(20)^. Zinc and folate are micronutrients that are essential for biological processes that support linear growth, including cellular metabolism and growth as well as protein synthesis^(58,59)^. Inadequacies in vitamin A are also known to correlate with impaired growth, given that deficiencies may limit cellular proliferation^(60)^. Furthermore, studies have shown that children with lower HAZ scores tend to have lower levels of PUFA and amino acids^(61)^, critical macronutrients that can be implicated by increased UPF consumption. Recognising the importance of these key nutrients for linear growth, the consistent findings of reduced intake among young children in Latin America could be extrapolated to our findings. The results in Table 3 do not suggest displacement of unprocessed food groups by UPF. Moreover, MDD scores for children in the highest tertile of UPF consumption were high enough to suggest adequate intakes of trace minerals or other nutrients required in small quantities^(62)^. However, these results, as well as the MDD scores, may not necessarily and adequately reflect dietary quality^(63,64)^ nor the consumption of other nutrients in sufficient amounts to support optimal linear growth.

Additional pathways that may shed some perspective on the observations for linear growth pertain to the impacts of UPF as a pro-inflammatory food class that can disrupt gut microbiota and intestinal integrity. During a child’s early years, a healthy gut microbiome is key for the prevention of local and systemic intestinal inflammation^(65)^. Intestinal inflammation impairs the absorption of critical nutrients that support linear growth. Moreover, poor diets that do not support microbiota diversity and microbial succession can increase the risk of enteropathy and diarrhoea, which significantly impair a child’s nutrient utilisation^(65)^.

A wealth of evidence has demonstrated negative effects of increased UPF intake on gut health. A study centred on a Spanish adult population found that gut microbiota richness and diversity were negatively correlated with increased UPF consumption in males^(24)^. The study also found an association between increased UPF consumption and the *Enterobacterales* bacterial order, which includes *E. coli.* Another study in mice found that a high-fat and high-sugar diet altered the gut microbiota and enhanced an inflammatory gastrointestinal environment with increased levels of mucosa that favoured *E. coli* colonisation in the gut^(23)^. *E. coli* and associated enteropathogenic outcomes during early childhood are consistently linked to diarrhoea, gastrointestinal inflammation and growth faltering^(66,67)^. Emulsifiers, an industrial ingredient found in many UPF^(12)^, have also been associated with negative impacts on gut microbiota composition and consequent colon and intestinal inflammation in mice^(68,69)^.

Although results from the univariate analysis assessing the relationship between caregiver-reported diarrhoea and UPF consumption were statistically non-significant, the prevalence of diarrhoeal morbidity within the highest tertile of UPF consumption was comparatively higher (26·8 %) than the prevalence for the lowest tertile of UPF consumption (16·7 %). It is therefore possible that the increased odds of stunting in the highest tertile may be mediated by UPF-triggered gut inflammation and related diarrhoeal outcomes.

Our study presented limited evidence for relationships between UPF consumption and increased WHZ, contrary to our hypothesis for weight status. Adjusted magnitudes of association generally trended in the negative direction, with savoury snacks being the only factor significantly associated with decreased WHZ. These findings did not parallel the general consensus around UPF consumption and its links to increased risk of overweight and obesity in children^(17,18)^. However, similar findings were reported in Pries and colleagues’^(22)^ study from Nepal, with non-significant and negative associations reported for associations between total energy intake from complementary ultra-processed snack foods and weight-for-length z-scores. Given the young ages of the children in the Lulun II cohort, it is likely that the cumulative effect of UPF on overweight outcomes is not yet present and may manifest later in life^(70)^.

WHZ is used to indicate overweight as well as wasting (WHZ < –2), an acute presentation of child undernutrition characterised by rapid weight loss in a child or their inability to gain weight^(71)^. Although there were no children with wasting nor overweight in the sample, the negative trends found across the models – particularly for the statistically significant salty snack category – may signal the potential for increased risk of acute weight loss. Here, the potential mechanism may, again, be related to the poor nutritional quality of UPF or the negative effects that excessive UPF intake induces on the gut microbiota. In children with acute undernutrition, having undernourished or immature gut microbiota is known to induce weight loss mainly due to disruptions in amino acid metabolism, which consequently leave the body in a catabolic state and hinder the building of lean mass^(65)^. It is imperative that future observational studies attempt to better understand potential mechanisms for this downward weight trend. This is particularly important for low- and middle-income settings where child wasting is prevalent, but UPF consumption continues to grow rampant.

Our findings on bone maturation did not support our established hypothesis nor the emerging evidence directly assessing UPF impacts on bone health parameters. Furthermore, given the significant positive association that has previously been found between BAZ and HAZ in this cohort^(26)^, we anticipated that the directions for the respective associations between UPF consumption and indicators of linear growth and bone maturation would be similar. One potential explanation for our findings on bone maturation pertains to the likely intake of Calcium, a mineral that supports bone ossification, from UPF. Despite evidence generally associating UPF consumption with reduced intake of several critical nutrients, some representative epidemiological studies have specifically observed increased intake of Calcium in relation to increased UPF intake^(34,72,73)^. It is however important to recognise that dietary patterns and nutrient interactions within a diet matrix – rather than a single nutrient – are better predictors for nutrition-related outcomes^(74,75)^. Emerging experimental evidence mostly points to the negative impacts of increased UPF intake on bone health. In one study, increased consumption of UPF was found to damage the growth plate zones in the bones of infant rats, suggesting increased risk for stunting^(25)^. Moreover, UPF intake had a negative impact on the genetic material that is involved in promoting bone quality and elongation^(25)^. In Travinsky-Shmul and colleagues’ study with rodent models^(76)^, a UPF-based diet was found to negatively impact several bone growth and quality parameters, with minimal improvements resulting from supplementation with Calcium or a multivitamin–mineral complex. The same study also found that a UPF-based diet increased bone marrow adiposity^(76)^. In the present study, the higher BAZ scores for children in the highest tertile may have been confounded by increases in bone marrow adiposity, possibly a result of UPF consumption patterns.

An additional mechanism through which UPF-induced bone advancement may occur is through inflammation. UPF consumption has been linked to an array of inflammatory biomarkers^(15)^, including IL-1*β* and IL-6^(77)^. In adults, the inflammatory cytokine IL-1*β* has been found to modulate endochondral ossification via the bone marrow^(78)^. On the other hand, inflammation may be associated with decreased bone age but through the pathway of impaired height velocity. In a study comparing young children with HIV according to the degree of height velocity impairment, the release of IL-6 was negatively correlated with bone age^(79)^. Western dietary patterns, characterised by significant UPF consumption, have been linked to increases in IL-6^(15)^. UPF-induced inflammation via IL-6 may therefore explain the significant findings observed for linear growth outcomes after controlling for child bone age (i.e. direct effect).

The significant and positive association found between UPF consumption and bone age in our study may suggest early bone maturation, which may not necessarily reflect the potential for sustained linear growth^(80,81)^. Bone age assessments in studies of older children, also following the standards of Greulich and Pyle^(39)^, have shown positive correlations between bone age and height during childhood^(80,82)^. While these associations parallel results previously reported for the cohort in this present study^(26)^, this observed association may present implications for the children’s growth later in life. Evidence suggests that young children with advanced bone ages reach their adult heights at a faster rate and would likely have sub-optimal heights as adults^(80,81)^. Studies citing this relationship between bone age and height have often also found positive associations between bone age and other outcomes that are typically associated with increased UPF intake including obesity, adiposity, hyperinsulinaemia and hyperthyroidism^(37,80,81)^. Given the absence of overweight in this sample, obesity is not a likely mediator of the significant relationship between UPF and bone age. On the other hand, body fat could have mediated this relationship. Body composition was not assessed in Lulun II. However, it is possible that some of the children with high BAZ scores may have had slightly higher body fat. Studies of children in Brazil have associated stunting with increased fat mass and central adiposity^(83,84)^. Given the high prevalence of stunting in the highest tertile of UPF consumption (61 %), we can infer that increased body fat may have been a key factor in our findings for bone maturation of children in the highest tertile of UPF intake. The significant relationship found between UPF intake and BAZ may also be through the pathway of other cardiometabolic biomarkers or outcomes which were generally beyond the scope of the larger Lulun study. These findings merit further research to identify mediating inflammatory and cardiometabolic biomarkers, particularly those that are equally implicated in UPF consumption and the outcomes of bone quality and stunted growth.

It is important to recognise that univariate analyses generally resulted in trending but non-significant associations between the frequency of UPF intake and the outcomes of interest. Thus, the findings presented here, particularly for linear growth, should be viewed in the context of covariate factors that were included in the analyses. Our findings suggest that UPF consumption influences the outcomes studied only after accounting for these factors. The literature generally suggests that the confounding exposures bear some influence on both UPF consumption and nutrition status. In emerging economies, higher-income households are more likely than poorer household to purchase UPF, whereas the opposite is observed for poorer households in high-income countries^(1)^. On the other hand, stunted growth is known to be more prevalent among poorer households in low- and middle-income countries^(85,86)^. Consumption of UPF also contributes to energy intake which also influences nutritional status. Dietary diversity, as a proxy for energy intake^(42)^ as well as food access and security^(87)^, may also be related to choices around UPF consumption. In some populations, food insecurity has been associated with increased intake of UPF^(88)^. Future research may consider studying socio-economic factors and measures of diet quality and access as effect modifiers in the relationship between UPF intake and health outcomes.

Analyses of a random convenience sample of UPF found in a primary school environment demonstrated the availability of unhealthy food items, as reflected in elevated contents of saturated fats, added sugars and sodium, along with low amounts of protein and fibre. The nutrition profiling presented here only represents a small sample of UPF available to children in the community. The results are, however, consistent with qualitative findings of a recent Ecuadorian study demonstrating child preferences for unhealthy UPF which are often obtained within the school environment^(89)^. The school food environment acts as a proxy for understanding potential UPF consumption trends among the younger children sampled, recognising the importance of likely food sharing with older siblings^(90)^. Studies from Latin America suggest that children are increasingly exposed to UPF through easy access within their homes and neighbourhoods^(91)^ as well as their school environments^(92–94)^. Further research directly linking child growth outcomes with UPF nutrition profiles and availability in children’s environments is needed.

This study bears limitations which should be considered when interpreting the findings reported here. Being a cross-sectional analysis, the temporal sequence between UPF consumption and each outcome studied is not clear. Thus, the results should be viewed merely as associations, rather than causal relationships, between the exposures and outcomes. Additionally, being based on a relatively small and rural population sample, the children sampled here may not be representative of different populations worldwide or in Ecuador. The operationalisation of UPF exposure also presents some limitations. Although the 24-hour FFQ is a widely used and validated technique for conducting dietary assessments, a single 24-hour FFQ does not adequately capture usual intake of UPF; it is, however, useful in describing average dietary intake in a population^(95)^. Additionally, given that we did not collect detailed dietary data to capture standard portion sizes and energetic intake, we were limited in our ability to determine the proportion of energy intake attributed to UPF consumption, which may influence the outcomes studied. Social desirability bias is also somewhat likely with food intake reporting using the FFQ. However, the questionnaire asked for consumption frequencies of different foods that have been found to be a part of the Ecuadorian diet^(29)^ and not necessarily for ‘UPF’. Despite these limitations, this study may be the first, to our knowledge, to assess the potential effects of different UPF on child growth, while attempting to elucidate the role that skeletal maturity plays in this hypothesised aetiology. Otherwise, prior evidence on our primary hypotheses has been based on experiments with rodent models.

### Conclusion

In Ecuador and elsewhere in Latin America, national dietary guidelines emphasise the importance of limiting UPF consumption^(11,89)^. These guidelines, as reflected in package labelling, often focus on consumer information highlighting overweight, obesity and other nutrition-related non-communicable diseases, while there is less emphasis on potential impacts on child undernutrition. However, through our study of a rural population in Ecuador (where stunting disproportionately affects rural and Indigenous populations^(6)^), we found that increased UPF consumption was associated with stunted growth. Considering the existing double burden of malnutrition globally and in Ecuador, where risk profiles for overweight and obesity may parallel those for undernutrition, this study provides key insights into UPF consumption as a potentially underreported risk factor for growth faltering. The findings presented here demonstrate the importance of understanding outcomes of undernutrition in research and discourse around UPF consumption. Policy considerations and dietary guidelines on UPF availability, access and consumption may highlight complex pathways from increased UPF intake to growth faltering as part of key strategies to holistically address poor nutrition across the malnutrition spectrum.

## Supplementary material

For supplementary material accompanying this paper visit https://doi.org/10.1017/S0007114523000624. click here to view supplementary material
