# Supplementary material for: Ultra-processed foods in a rural Ecuadorian community: associations with child anthropometry and bone maturation
Source: Br J Nutr. 2023 Mar 13;130(9):1609–24. doi: 10.1017/S0007114523000624 (PMC10551472; doi:10.1017/S0007114523000624)
Supplement: Supplementary file 1 [file S0007114523000624sup001.docx]

**Ultra-processed foods in a rural Ecuadorian community: associations with child anthropometry and bone maturation**

**SUPPLEMENTARY DATA**

**Supplementary Figure 1**

| **Male at 32 months** | **Female at 36 months** |
| --- | --- |
| **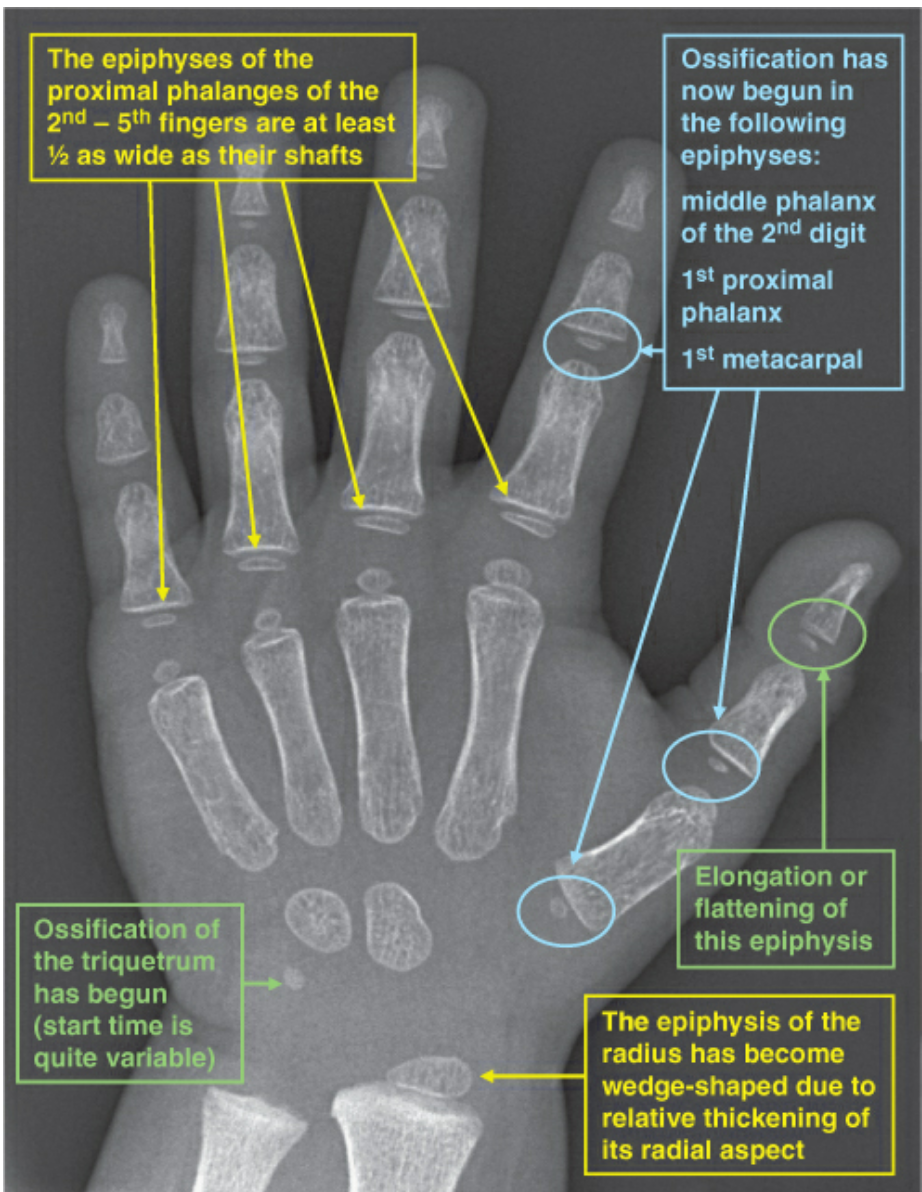** | **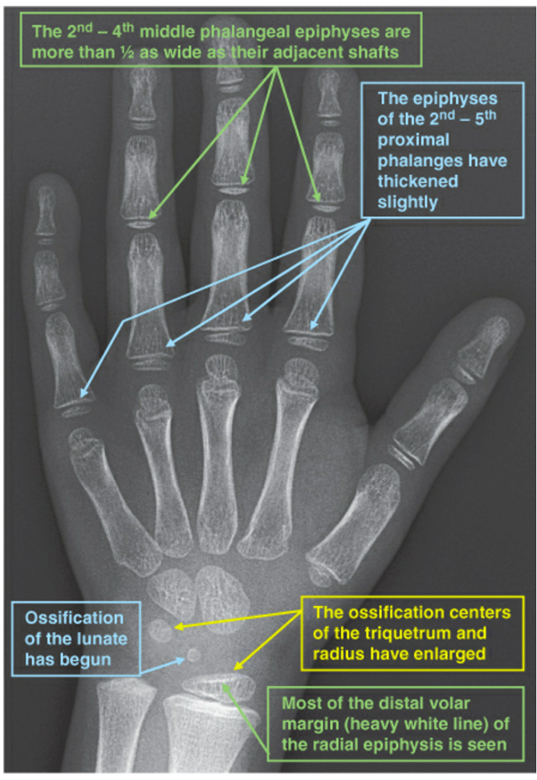** |

Images from: Gaskin CM, Kahn MM, Bertozzi JC, Bunch PM. Skeletal development of the hand and wrist: a radiographic atlas and digital bone age companion. Oxford University Press; 2011 Feb 1. Used with permission from publisher.

**Supplementary Table 1: Ossification centers evaluated by age and sex of child based on standards of Greulich and Pyle**

| **Age (months)** | **Male Child** | **Female Child** |
| --- | --- | --- |
| 3 | - Capitate - Hamate | - Capitate - Hamate |
| 12 | No new ossification | - Distal radial epiphysis - Second metacarpal - Second proximal phalanx - Third metacarpal - Third proximal phalanx - Fourth proximal phalanx |
| 15 | - Distal radial epiphysis | - Fourth metacarpal - Thumb distal phalanx |
| 18 | - Second metacarpal - Second proximal phalanx - Third proximal phalanx - Fourth metacarpal - Fourth proximal phalanx - Thumb distal phalanx | - Fifth metacarpal - Fifth proximal phalanx |
| 24 | - Fifth metacarpal - Fifth proximal phalanx - Third middle and distal phalanx - Fourth middle and distal phalanx | - Triquetrum - Thumb metacarpal - Thumb proximal phalanx |
| 30 | NA | - Second distal phalanx - Fifth middle phalanx |
| 32 | - Thumb metacarpal - Thumb proximal phalanx - Triquetrum - Second middle phalanx - Second distal phalanx - Fifth distal phalanx | NA |
| 36 | - Lunate | - Lunate |
| 42 | No new ossification, but size and morphology of second distal phalanx and fifth distal phalanx were evaluated | - Trapezium |
| 48 | - Trapezium, all phalanges (evaluated for size and morphology) | NA |
| 50 | NA | - Scaphoid - Trapezoid |

**Supplementary Table 2: Bone-age mediated relationships between UPF consumption and linear growth indicators**

| **UPF Intake** | **Direct effect (Bootstrap 95% CI)** | **P value**^a^ | **Indirect effect**  **(Bootstrap 95% CI)** | **P value**^a^ |
| --- | --- | --- | --- | --- |
| **HAZ** | | | | |
| **Tertile 1** | Reference | | |  |
| **Tertile 2** | 0.08  (-0.28—0.44) | **0.04** | 0.09  (-0.01—0.19) | 0.15 |
| **Tertile 3** | -0.36  (-0.74—0.02) |  | 0.11  (-0.01—0.23) |  |
| **Stunting** | | | | |
| **Tertile 1** | Reference | | |  |
| **Tertile 2** | 0.09  (-0.85—1.04) | 0.07 | -0.21  (-0.46—0.04) | 0.23 |
| **Tertile 3** | 1.07  (0.06—2.07) |  | -0.25  (-0.56—0.06) |  |

^a^ Wald Chi-squared test

* Bolded *P values* indicate significance for Wald Chi-squared test**.**

**Supplementary Table 3: List of ultra-processed foods sampled and summary of individual nutrition profiles**^a^

| **ID** | **Product name** | **Energy, kJ** | **Energy, kcal** | **Saturated fat, g** | **Energy from saturated fats, kcal** | **Free sugars, g** | **Energy from free sugars, kcal** | **Sodium,**  **mg** | **Fiber, g** | **Protein, g** | **Percentage of whole fruit, vegetables, or nuts^b^** |
| --- | --- | --- | --- | --- | --- | --- | --- | --- | --- | --- | --- |
| 1 | Sorbetitos Sami^‡^ | 1680 | 401.52 | 0 | 0 | 100 | 400 | 0 | 0 | 0 | 0 |
| 2 | Doritos Lemon Remix | 2096.7 | 501.1113 | 9.99 | 89.91 | 0 | 0 | 765.9 | 0 | 6.66 | 0 |
| 3 | Gelactica (Gomitas de Frutas/Jelly Fruit) ^‡^ | 420 | 100.38 | 0 | 0 | 25 | 100 | 0 | 0 | 0 | 0 |
| 4 | Recontra Acido boca loca (Red) ^‡^ | 840 | 200.76 | 0 | 0 | 20 | 80 | 0 | 0 | 0 | 0 |
| 5 | Sabor Uva Lengua^‡^ | 1260 | 301.14 | 0 | 0 | 75.3 | 301.2 | 20 | 0 | 0 | 0 |
| 6 | Chicle perlitas acido^‡^ | 1260 | 301.14 | 0 | 0 | 80 | 320 | 0 | 0 | 0 | 0 |
| 7 | Paletaufo Krazymon Maracuyá con Sal y Lemon^‡^ | 1260 | 301.14 | 0 | 0 | 50 | 200 | 10600 | 0 | 0 | 0 |
| 8 | Papa Nic Maracuya | 1726.1 | 412.5379 | 0 | 0 | 0 | 0 | 2735.2 | 10.52 | 15.78 | 0 |
| 9 | Papa Nic Limon | 1465 | 350.135 | 0 | 0 | 0 | 0 | 2700 | 10 | 15 | 0 |
| 10 | Recontra Acido boca loca (yellow)^‡^ | 840 | 200.76 | 0 | 0 | 20 | 80 | 0 | 0 | 0 | 0 |
| 11 | Chicle perlitas^‡^ | 1260 | 301.14 | 0 | 0 | 80 | 320 | 0 | 0 | 0 | 0 |
| 12 | Gelatina Sabor a frutas^‡^ | 24.31 | 5.81009 | 0 | 0 | 0 | 0 | 0 | 0 | 0 | 0 |
| 13 | Paletaufo Krazymon Mango con Sal y Lemon^‡^ | 1260 | 301.14 | 0 | 0 | 50 | 200 | 10600 | 0 | 0 | 0 |
| 14 | Los Habiteñitos Habitas ^c^ | 2510 | 599.89 | 10 | 90 | 0 | 0 | 550 | 20.5 | 20 | 0 |
| 15 | Nucita (Crema con sabor a leche, chocolate y nueces) ^‡^ | 2138 | 510.982 | 4.44 | 39.96 | 60 | 240 | 122 | 0 | 2.22 | 0 |
| 16 | Salticas Galletas Salatidas | 2094.6 | 500.6094 | 8.33 | 74.97 | 6.66 | 26.64 | 799 | 0 | 3 | 0 |
| 17 | TaniLact^d‡^ | 408.1 | 97.5359 | 1.59 | 14.31 | 8.48 | 33.92 | 42.4 | 0 | 2.12 | 0 |
| 18 | Colombina Piazza (barquillos Sabor a malteada de fresa) ^‡^ | 2108.7 | 503.9793 | 11.6 | 104.4 | 38.8 | 155.2 | 78 | 3.9 | 3.88 | 0 |
| 19 | Circus Galletas con crema sabor a fresa^‡^ | 1945.7 | 465.0223 | 8.93 | 80.37 | 32.1 | 128.4 | 410.6 | 0 | 3.6 | 0 |
| 20 | Topsy choc ice cream^‡^ | 500.5 | 119.6195 | 4.59 | 41.31 | 8.25 | 33 | 12.87 | 0 | 0 | 0 |
| 21 | Yellies Marshmallows (helados rellemos)^e‡^ | 1260 | 301.14 | 0 | 0 | 60 | 240 | 0 | 0 | 0 | 0 |
| 22 | Tango Báilate (Sabor a chocolate)^f‡^ | 2180 | 521.02 | 12 | 108 | 44 | 176 | 160 | 1.6 | 4 | 0 |
| 23 | Pipa Chos | 2725 | 651.275 | 5 | 45 | 0 | 0 | 250 | 5 | 15 | 0 |
| 24 | Led Chupi Tops^‡^ | 1400 | 334.6 | 0 | 0 | 62.5 | 250 | 37.5 | 0 | 0 | 0 |
| 25 | Golpe Oblea Rellena Cubrieta con Caramelo y Cereal Crocante^‡^ | 2171.9 | 519.0841 | 11.1 | 99.9 | 59.2 | 236.8 | 111 | 3.7 | 3.7 | 0 |
| 26 | Orbita Galettas Rellenas con Crema Sabor a vanilla con Cobertura sabor a chocolate^‡^ | 2012 | 480.868 | 16 | 144 | 36 | 144 | 120 | 0 | 4 | 0 |
| 27 | Frunas Frutas^‡^ | 354 | 84.606 | 2.08 | 18.72 | 56.2 | 224.8 | 0 | 0 | 0 | 0 |
| 28 | Colombina Max (Frutacidas) ^‡^ | 353 | 84.367 | 0 | 0 | 64.7 | 258.8 | 59 | 0 | 0 | 0 |

^a^ All quantities are per 100g of product sample.

^b^ Per UK NPM guidelines, excludes ***seeds*** which are not explicitly considered as nuts. Product items 14 and 23 include whole seeds—fava beans and sunflower seeds, respectively.

**^c^** Sugar and fiber content based on USDA estimates for fiber and sugar in fava beans, the primary ingredient for this product.

^d^ Values adjusted for the density of yogurt (1.06 g/ml).

^e^ Fat content based on USDA estimates for fat in marshmallows, the primary ingredient for this product.

^f^ Fiber content based on USDA estimates for fiber in a generic chocolate wafer.

^‡^ Indicates sugary/sweetened snack
